# Is health politically irrelevant? Experimental evidence during a global pandemic

**DOI:** 10.1136/bmjgh-2020-004222

**Published:** 2020-10-23

**Authors:** Arnab Acharya, John Gerring, Aaron Reeves

**Affiliations:** 1 Independent Researcher, Washington, DC, UK; 2 University of Texas, Austin, Texas, USA; 3 Department of Social Policy and Intervention, University of Oxford, Oxford, Oxfordshire, UK

**Keywords:** public health, health policy, individual randomized trial

## Abstract

**Objective:**

To investigate how health issues affect voting behaviour by considering the COVID-19 pandemic, which offers a unique opportunity to examine this interplay.

**Design:**

We employ a survey experiment in which treatment groups are exposed to key facts about the pandemic, followed by questions intended to elicit attitudes toward the incumbent party and government responsibility for the pandemic.

**Setting:**

The survey was conducted amid the lockdown period of 15–26 April 2020 in three large democratic countries with the common governing language of English: India, the United Kingdom and the United States. Due to limitations on travel and recruitment, subjects were recruited through the M-Turk internet platform and the survey was administered entirely online. Respondents numbered 3648.

**Results:**

Our expectation was that respondents in the treatment groups would favour, or disfavour, the incumbent and assign blame to government for the pandemic compared with the control group. We observe no such results. Several reasons may be adduced for this null finding. One reason could be that public health is not viewed as a political issue. However, people do think health is an important policy area (>85% agree) and that government has some responsibility for health (>90% agree). Another reason could be that people view public health policies through partisan lenses, which means that health is largely endogenous, and yet we find little evidence of polarisation in our data. Alternatively, it could be that the global nature of the pandemic inoculated politicians from blame and yet a majority of people do think the government is to blame for the spread of the pandemic (~50% agree).

**Conclusions:**

While we cannot precisely determine the mechanisms at work, the null findings contained in this study suggest that politicians are unlikely to be punished or rewarded for their failures or successes in managing COVID-19 in the next election.

**Trial registration:**

Initial research hypotheses centred on expected variation between two treatments, as set forth in a detailed pre-analysis plan, registered at E-Gap: http://egap.org/registration/6645. Finding no difference between the treatments, we decided to focus this paper on the treatment/control comparison. Importantly, results that follow the pre-analysis plan strictly are entirely consistent with results presented here: null findings obtained throughout.

**Key questions**

**What is already known?**

- Political leaders in democracies are sensitive to cues from the electorate and are less likely to implement unpopular policies.
- Electoral accountability is not automatic, however, and it only exists if citizens connect specific policies to politicians and vote accordingly.
- We know little about how public health attitudes affect voting intention or behaviour.

**What are the new findings?**

- The majority of our respondents believe health is an important policy area and that government has some responsibility for health.
- Most of our respondent think their government is to blame for the spread of the pandemic.
- However, we find that those exposed to key facts about the pandemic are no more likely to favour, or disfavour, the incumbent nor to assign blame to government for the pandemic compared with an untreated control group.

**What do the new findings imply?**

- It is unclear whether politicians will be punished or rewarded for their failures or successes in managing COVID-19 in the next election.
- Our results suggest that democracies may not improve health because of electoral accountability as is commonly assumed but perhaps for other reasons.
- While we are cautious about drawing strong conclusions from a single experiment, our results also speak to how political institutions might contribute toward the sustainable development goals.

If public health policies are to succeed they must receive support from the people they are designed to help. This is especially true in democracies, where politicians are subject to periodic election and therefore sensitive to cues from the electorate. However, electoral accountability is not automatic; it exists only if citizens connect specific policies to politicians and vote accordingly. Do issues surrounding public health move public opinion and do those opinions translate into voting behaviour?

We know a fair bit about attitudes toward public health in rich countries, where health systems are expansive and expensive, and especially in the United States, where healthcare is an intensely partisan issue.^1–13^ We know less about attitudes toward public health in the developing world, where the topic is rarely studied.^14^ In neither context is the opinion/behaviour nexus well understood. Although a few studies examine the association between public health attitudes and voting behaviour,^15^ it is difficult to infer causality from such observational data.

The COVID-19 pandemic offers a unique opportunity to observe the interplay between public opinions about public health and electoral politics. This pandemic is one of the worst of the modern era; it is global in scope; it is covered intensively by the press; and the role of political leaders and parties in mitigating or exacerbating the pandemic is front-and-centre in news reportage. If public health matters for popular politics, COVID-19 would seem to be a perfect storm.^16^

To assess the question, we launched surveys in the United States, the United Kingdom and India. Embedded within the surveys is a survey experiment in which we prime information about the health and economic effects of the COVID-19 pandemic.

## Research design

The COVID-19 pandemic is global in reach, which means mass publics and politicians around the world are confronted with similar challenges. Political responses differ, and exposure to the virus also differs across countries. However, all citizens share the uncertainty of knowing that a massive number of deaths—beyond anything experienced over the past century—is possible. In this general sense, the COVID-19 threat is ubiquitous.

To gauge public opinion across the world an ideal research design would incorporate random samples drawn from every country at regular intervals. We do not have the resources or the logistical wherewithal to carry out such a massive undertaking. Nor is it possible in the midst of this highly contagious pandemic to administer surveys person-to-person, which impedes the ability to recruit random samples in many countries. For our purposes, it is also important to capture opinion in a country at a point in time when the pandemic (as judged by infection and mortality rates) and public attention to it (as judged by popular media accounts) is near its peak. We cannot wait for the pandemic to subside in order to contact research subjects in a safe environment.

Accordingly, we employ a survey recruitment platform that is widely used for survey experiments and which has a significant presence in select countries around the world: Amazon’s Mechanical Turk (‘M-Turk’).^17^ For theoretical reasons, it is vital to include both more and less affluent countries. Accordingly, we selected countries that varied according to economic development and also provide a significant contingent of M-Turk workers^18^: the USA, the UK and India.

Power analyses suggested a sample size of 1500 in each country; that is, 500 for each arm of the experiment. We were cognisant that the smaller numbers of Turkers in the UK might preclude reaching a full sample in that country. However, the more important issue was obtaining sufficient samples in the developed world (for which the UK and USA could be considered together) and the developing world (for which India would have to suffice).

Recruitment took place over several weeks, from 15 April to 6 June 2020. The US quota was filled within a few days, the Indian quota took nearly 2 months, and the UK quota was not entirely met (n=615). Accordingly, respondents were reached at different points in time in India and the UK, offering information about the stability of responses. The survey was available in English for all three countries and in English and Hindi for India. Survey respondents were compensated through M-Turk according to rates that account for differences in purchasing power parity across the three countries.

The resulting sample is younger, includes more men, and is more educated than the general populations of the three countries—a common pattern among M-Turk studies.^18^ The India sample is also more urban and better off than the general population. Additionally, there are some regional imbalances, with London over-represented in the UK and southern states over-represented in the Indian sample. Further details are provided in online supplemental appendix B.

10.1136/bmjgh-2020-004222.supp1 Supplementary data

To ascertain how the COVID-19 pandemic might influence political behaviour we employ a survey experiment in which key facts about the pandemic are revealed to respondents, followed by questions intended to elicit attitudes toward the government and potential voting choices (this follows a widely employed technique known as the survey experiment^19^). The first treatment deals with the possible economic impact of the pandemic and the second concerns its possible health impact. A filter question ensures that respondents comprehend the information that has been presented to them and also serves to reinforce the initial stimulus. Thus, we construct an experiment with two treatment groups and a control group (which is given no information about the pandemic). Outcome questions of theoretical interest inquire (a) whether respondents would support the incumbent (party and party leader) if an election were held today, and (b) whether they hold the government at fault for allowing the disease to spread. Further details on the setup are contained in box 1 and a complete questionnaire is provided in online supplemental appendix A.

Box 1 **Experimental design**

The experiment consists of a pure control and two treatments. The first treatment focuses on the projected economic effects of the COVID-19 pandemic. The US version reads as follows:

As you are probably aware, the Coronavirus disease (COVID-19) has spread around the world. Experts are wrestling with the impact of this pandemic on the United States economy. Some estimates suggest that the economy could shrink by 3.2% this year, that 52.8 million people could end up without work (around 32% of the entire workforce), and that the value of stocks and shares could fall by around 30%.

Similar versions are constructed for the UK and India based on economic projections in those countries.

The second treatment focuses on the health effects of COVID-19. The US version reads as follows:

As you are probably aware, the Coronavirus disease (COVID-19) has spread around the world. Experts are wrestling with the impact of this pandemic on public health in the United States. One estimate suggests that around 12.9 million would require hospitalisation (3.9% of the population), around 3.7 million would need critical care, and over 2.8 million people could die (around 0.8% of the population). At present, there is no vaccine for Coronavirus and no cure.

Similar versions are constructed for the UK and India based on health projections for the virus in those countries.

After each treatment, the respondent is asked a multiple-choice question about the information presented in the previous page. For example, after the health treatment US respondents might be asked:

What is the estimated number of fatalities from COVID-19 in the USA, as stated on the previous page? (If you are not sure, check back on the previous page.)

- It was more than 2 million people
- It was less than 2 million people

Follow-up questions are constructed so that the correct answer is the highest—(a) rather than (b)—so as to enhance the strength of the treatment. Respondents must answer this follow-up question correctly in order to proceed through the survey.

Two outcome questions gauge the possible impact of these treatments on political behaviour. The first centres on the incumbent: If a national election were held today, would you like to see [Johnson and the Conservative party/Trump and the Republican party/Modi and the BJP] reelected? The second asks whether the government is at fault for allowing the pandemic to spread. Responses are registered on a 100-point feeling thermometer.

Two hypotheses will guide our discussion. (Initial research hypotheses centred on expected variation between the two treatments, as set forth in a detailed pre-analysis plan, registered at E-Gap: http://egap.org/registration/6645. Finding no difference between the treatments, we decided to focus this paper on the treatment/control comparison. However, it is important to note that results from the pre-analysis plan are entirely consistent with results presented here: null findings obtained throughout.)

*H_1_:* Respondents who receive either of the treatment conditions will offer greater—or lesser—support for the incumbent relative to the control group.

*H_2_:* Respondents who receive either of the treatment conditions will be more inclined to blame the government for their handling of the pandemic.

To test these hypotheses, we adopt an experimental design that contrasts two treatment conditions with the control condition, as described. We estimate causal effects through the following regression model, which includes background covariates in order to yield greater precision:

$$Outcome_{i}=constant+\alpha SES_{i}+\beta C+\gamma T_{i}+\delta Country_{i}$$

where Outcome is the measurement of the response to the scenario to which the person i was exposed, denoted by T, SES denotes the socioeconomic factors, C stands for demographic factors, and Country represents dummies for the three countries.

## Results

To ascertain whether the experiment is effective in priming attitudes we ask several questions. First, we inquire about the (subjective) importance of two prominent policy areas: public health and the economy. Each is gauged on a 100-point feeling thermometer. An index is then constructed by subtracting views on the importance of public health from views about the importance of the economy (which functions as a baseline).

Second, we ask about worries with respect to the health and economic effects of the COVID-19 pandemic. Again, we compose an index by subtracting worries about health from worries about economics (which functions as a baseline).

**Figure 1:**
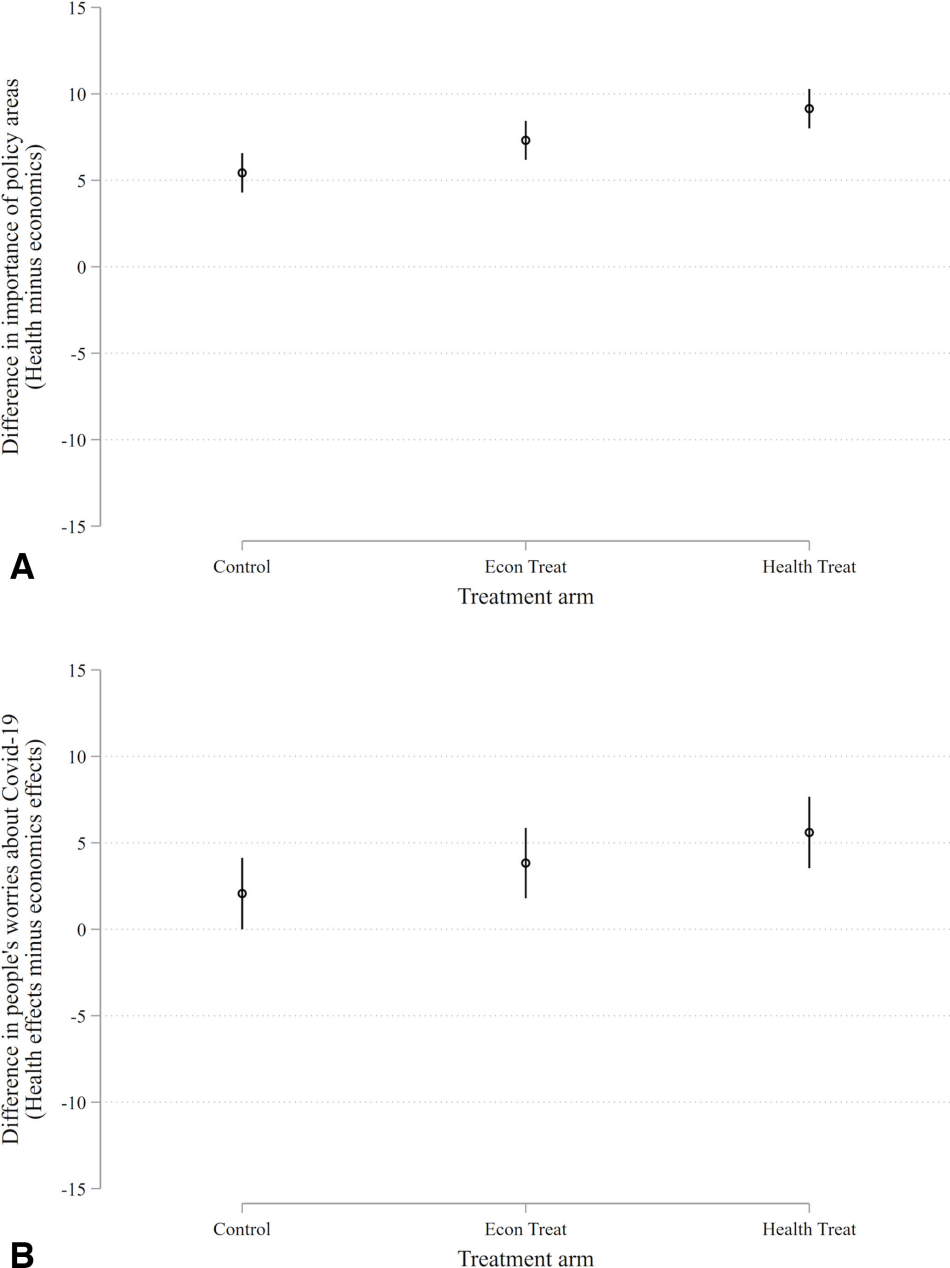
(A) Salience. Point estimates represent marginal effects and vertical bars are 95% confidence intervals. Each person is asked about how important they think each policy (health and economy) is on a scale from 0 to 100 (100=very important). We then calculate the difference (health – economy). The full scale of this measure ranges from −100 to 100. Economic condition versus control: β=1.9, p=0.022; health condition versus control: β=3.7, p<0.001. (B) Worry. Point estimates represent marginal effects and vertical bars are 95% confidence intervals. Each person is asked about how worried they are about the economic and health effects of COVID-19 on a scale from 0 to 100 (100=very worried). We then calculate the difference (health – economy). The full scale of this measure ranges from −100 to 100. Economic condition versus control: β=1.8, p=0.234; health condition versus control: β=3.5, p=0.018.

Results of these analyses, shown in figure 1A, B, indicate that both treatment conditions boost the salience of public health relative to the economy and make people more worried about the health effects of COVID-19. Moreover, the pure health treatment produces a stronger effect than the treatment focused on the economic effects of COVID-19, as one would expect if the experiment is having the intended effect.

**Figure 2:**
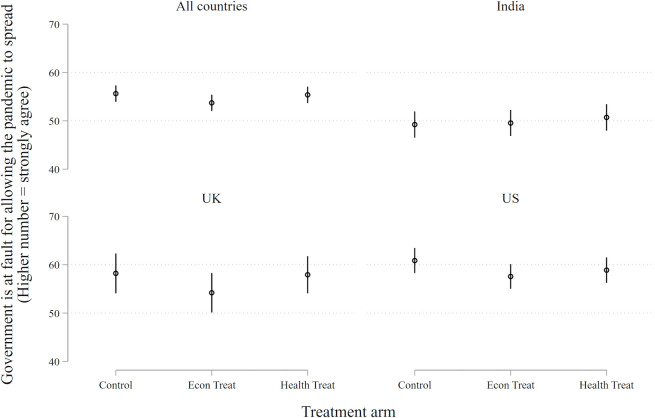
Incumbent support. Point estimates represent marginal effects and vertical bars are 95% confidence intervals. Each person is asked about how they would feel if the incumbent in each country was reelected on a scale from 0 to 100 (100=very happy). None of the treatment conditions in any country has a p-value less than 0.1.

**Figure 3:**
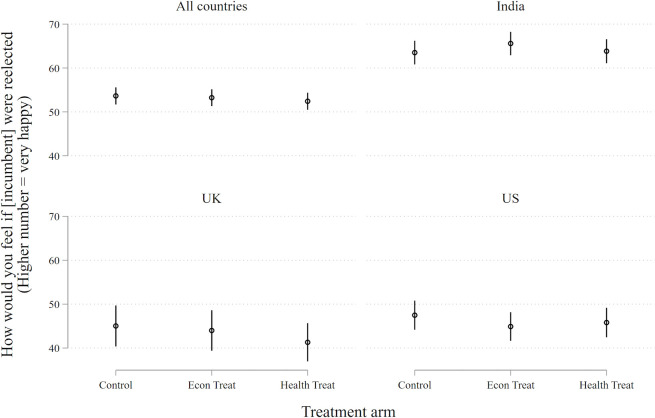
Government at fault. Point estimates represent marginal effects and vertical bars are 95% confidence intervals. Each person is asked about whether the government is at fault for the spread of COVID-19 on a scale from 0 to 100 (100=strong agreement). For none of the treatment conditions is p<0.05 and in only one is p<0.1.

Our theoretical interest is not salience or anxiety. We want to know whether the COVID-19 pandemic has *electoral* repercussions. For this to occur, concern about the virus must affect views about the ruling party and the sitting government, as contained in our two outcome questions (see box 1). These tests are presented in figures 2 and 3).

Neither treatment demonstrates an appreciable effect on either outcome. This generates four null results—a consistent pattern in which treatments designed to heighten the subject’s awareness of the economic and health dangers of the COVID-19 pandemic fail to change their support for the ruling party or their inclination to place blame at the feet of the government.

Granted, if the Indian sample is excluded from the analysis shown in figure 3, those subjected to the economic treatment are slightly *less* likely to blame their governments (economic condition vs control: β=−3.5, p=0.027). We are not sure what to make of this small effect on a subsample of respondents. Perhaps the effect means that the health pandemic has reinforced the standing of ruling parties in the USA and the UK, a result that runs against the grain of reportage (which has generally blamed these governments for their tardy response). In any case, it is a very small effect and is not robust in the full sample. We are inclined to regard it as stochastic.

## Interpretations

Null results are often more difficult to interpret than positive or negative results, so we shall spend some time considering what the findings represented in figures 2 and 3 might mean. Do they mean that the COVID-19 pandemic has no political consequences, as we have suggested, or is there some other explanation?

The experiment primes an existing condition that is well known to the participants, and is also highly salient. Under the circumstances, it may be difficult to move the dial, as research subjects are already saturated with information. However, the results shown in figure 1 demonstrate that the experiment did affect salience and anxiety (when public health is gauged against economics); this serves as a negative placebo, showing that subjects were attentive to the treatments and responsive to them. More generally, we doubt that the fear and uncertainty surrounding COVID-19 is so great that it would resist a further stimulus, provided by our—very pointed—reminder of its possible consequences. Arguably, it should be the reverse. If people are already aware of a problem, a reminder of that problem should stimulate those concerns by making them manifest. (By contrast, a less well known or less virulent health problem might not be perceived by subjects as a credible threat.)

A null finding may also result if respondents hold an extreme position, as measured by the outcome; they are at one end or the other. For such ‘extremists’ there may be no way to measure the impact of the treatment. This concern is mitigated by our response variable, a 100-point feeling thermometer. Still, 27% of our sample (987 of 3648) sit at the extremes of the scale (at 0 or 100) for the incumbency question (figure 2), and 11% of the sample (391 of 3648) sit at the extremes of the scale for the government-at-fault question (figure 3), as shown in Figure B3. A simple expedient is to remove the extremes from the analysis. Doing so reveals a treatment effect (for both the economics and health treatment conditions) that is often closer to zero than what is recorded in figures 2 and 3. Our null effects do not appear to be driven by a censored scale.

Polarised respondents are also more likely to have stable opinions irrespective of their exposure to the treatment and this may push our results towards the null. We explore whether moderates are responding to our treatments by first identifying those in the control group with less polarised views. Then we use a matching procedure to identify those in the treatment groups who are similar to—according to a set of background characteristics—those in the control group with these moderate views. Our matching procedure then excludes those in the treatment groups who are similar to those in the control group with extreme views. This analysis shows that even when we focus on those likely to hold moderate views on the outcomes of interest we still find null effects (see online supplemental figure C1 and accompanying text).

A null finding may also result from causal heterogeneity, when a treatment has disparate effects – sometimes positive, sometimes negative – on subjects depending on their background conditions. One obvious background condition is the country context. One can easily imagine that the COVID-19 pandemic might be experienced differently in the USA, the UK and India. However, figures 2–3 show that this is not the case.

Another background condition is partisanship. It is possible that our experimental prime has the effect of polarising respondents, making supporters of the incumbent even more supportive and opponents even more opposed, culminating in a null average effect. If this were the case, we would expect greater variance in the treatment groups than in the control groups for each country. We find no such differences, as measured by standard deviations.

Of course, background conditions of individual subjects are, in principle, infinite. Our post-survey questionnaire inquired about sex, age, urban/rural location, employment status, educational attainment, income, money saved for emergencies, and current health status. Split-sample tests focusing on subjects who fall into different categories along these various dimensions do not reveal any significant effects (see online supplemental table C1, C2).

## Generalisability

With experiments there are often questions about generalisability. In the present instance, one may wonder whether results contained in figures 2 and 3 are indicative of the outcome of theoretical concern—election-day behaviour.

With ‘positive’ treatment effects, generalisability would be more of a concern. After all, talk is cheap: responses to a survey have no consequences for the respondent, while in an election there is something important at stake. But there is no reason to suppose that the cheapness of talk would be conducive to null results. If anything, the reverse seems more likely.

A second concern is the time separation between our experiment and the arrival of the next national elections, which are not imminent in any of the studied countries (half a year in the USA and several years in the UK and India). Since public sentiments wax and wane according to many factors that are impossible to predict, effects associated with COVID-19 uncovered in the midst of a pandemic may dissipate once the pandemic subsides. However, dissipation also seems more of a problem for a ‘positive’ finding than a null finding. There is little reason to suppose that the political ramifications of COVID-19 would *increase* between now—the height of the crisis (or nearly so) in the three countries under observation—and the next election.

A third issue concerns the specific point in time that we chose for our experiment—late April to early May 2020. Perhaps there was something specific about that point in time, close to the apex of the pandemic, that engendered a null result from our respondents. Some leverage on this question can be garnered from the duration of the recruitment period, which lasted for 3 weeks in the UK and India. This means that our sample from these countries captures the state of the pandemic, and of politics, at somewhat different moments in time. During this period the total number of deaths in the UK rose from ~13 000 to~40 000, while India’s deaths rose from 405 to over 7000. When we compare responses gathered at different points in time we find a marked increase in the number of Indian respondents (across all treatment arms) who are inclined to blame their government, though there is no change among Indian respondents in their support for the incumbent’s reelection. UK respondents show no changes in their overall responses to either question. Most important, the estimated treatment effects for Indian and UK samples are null across various points in time, as shown in online supplemental figure C2. Accordingly, there is no indication that the results reported here are specific to a particular moment in time.

A fourth issue concerns the representativeness of our sample of M-Turkers. In addition to problems of self-selection, M-Turkers differ along standard demographic dimensions when compared with national populations, as noted. Some of them may be ‘professional’ survey respondents, and many (about three-quarters) have participated in previous surveys about COVID-19. To check whether these factors influenced the receptiveness of our sample to the experimental treatments we replicated the analyses pictured in figures 2–3 across subgroups—defined by those who have, or have not, taken previous surveys related to COVID-19 (according to self-report). Null results were obtained among each subgroup.

Another issue of generalisability concerns our choice of study sites. Are null results in the USA, the UK and India likely to be replicable in other contexts? This is much harder to assess. However, the USA and the UK are among the countries most affected by the COVID-19 health pandemic; in this respect their background circumstances are propitious. That India had been less affected (as of May 2020) offers a point of contrast. That the null finding persists across all three contexts suggests that these results might be generalisable.

Our three research sites also offer variation on another background condition of potential importance. In the USA, the COVID-19 pandemic has been subject to partisan politics, with a president who downplays its seriousness and has been widely faulted for a weak and inconsistent response to the public health threat. In the UK and India, partisan politics have also been at play, but not in such a prominent fashion. Again, it should be stressed that all three countries register a robust null result.

One may wonder whether findings with respect to this pandemic are generalisable to other health crises such as HIV/AIDS and Ebola. We are not aware of similar survey experiments conducted in the midst of these epidemics so it is difficult to speculate on this point. Likewise, one may wonder whether ‘normal’ public health issues such as the perennial tussle over government’s role in healthcare, the performance of government health services, and their expense would elicit similar responses. It is possible that the extreme nature of COVID-19, and its seemingly irresistible global spread, have inoculated politicians from blame. Further research will be needed to determine whether more mundane public health issues carry a stronger political punch.

## Discussion

People in the USA, the UK and India are extremely concerned about the pandemic—both its health effects and economic repercussions—and they become even more concerned when primed with information about the repercussions of the virus (see figure 1). Yet, we find no evidence that these worries translate into changes in political behaviour (see figures 2–3).

Several reasons may be adduced for this (unexpected) null finding. One reason could be that public health is not viewed as a political issue but rather as a matter of personal conduct, group status or socioeconomic standing. Another reason could be that members of the public view public health policies through partisan lenses, which means that health is largely endogenous. (It is worth noting, however, that a recent survey experiment situated in the USA found no impact on attitudes toward COVID-19 when partisan cues were primed.)^16 20^ Alternatively, it could be that the global nature of the COVID-19 pandemic has inoculated politicians from blame.

Whatever mechanisms might be at work, the null findings of this study suggest that politicians are unlikely to be punished or rewarded for their failures or successes in managing COVID-19 in the next election. One is tempted to conclude that public health issues have little influence on voter preferences in most election cycles. For example, it is not clear whether the stagnation of life expectancy in the USA and the UK, and the low level of public sector health expenditure in India have had much impact on recent elections in those countries. The urgency by which the COVID-19 pandemic has ripped through social, economic and political landscapes may challenge these complacencies, but only if mass publics make connections between the state of public health and what public officials can do. We need more research on how these factors interact, and the extent that they are disconnected, to determine why this might be so.

If public health is politically inconsequential this also raises questions about the impact of political institutions on health outcomes. Most studies suggest a positive relationship between democracy and improved public health proxied by mortality.^21–26^ Generally, this is attributed to electoral accountability.^27 28^ Democracies hold free and fair elections and these institutions make politicians more responsive to the preferences of citizens, which are thought to prioritise health.^24 29–31^ Our results suggest that this commonsensical argument may be flawed. Democracies may promote health, but perhaps for reasons other than electoral accountability. This accords with recent work that questions the viability of accountability as a mechanism of good governance^32^ or suggests alternative mechanisms such as the selection of good leaders.^33^

There are many potential implications from this study. At the same time, we want to caution against drawing big conclusions from a single experiment conducted in an exploratory manner. We noted potential problems of generalisability in the previous section. In particular, our M-Turk samples are not randomly drawn from their respective populations; it is possible that different results would be obtained with randomly chosen samples. There is also a question about our choice of country cases; only three countries were included and their representativeness of the world of nation-states could be questioned. Finally, there is a question about the policy itself; there may be features of COVID-19 that do not generalise to other health outcomes. These issues warrant further research.
