# Supplementary material for: Is health politically irrelevant? Experimental evidence during a global pandemic
Source: BMJ Glob Health. 2020 Oct 23;5(10):e004222. doi: 10.1136/bmjgh-2020-004222 (PMC7590354; doi:10.1136/bmjgh-2020-004222)
Supplement: Supplementary data [file bmjgh-2020-004222supp001.pdf]

Appendix A: Questionnaire

What follows is the questionnaire used for the United States. Similar questionnaires were devised for the United Kingdom (in English) and India (in English and Hindi).

Preliminary

Q1 You are being invited to participate in a research study conducted by Aaron Reeves from the University of Oxford. This study is being conducted in a number of countries and you have been invited to participate because you live in the United States.

Why are we doing this research study? The purpose of this research study is to understand your opinions about current events, and how they are affecting our lives.

Who can participate in this research study? Anyone can participate in this study as long as they are from US.

What will I be asked to do and how much time will it take? If you agree to take part in this study, you will be asked to complete a short online survey. This survey will ask about your knowledge of and opinions about current events, and it will take you approximately 5 minutes to complete.

What are my risks of being in this research study? There are no risks to you by participating this research. There will be no inconveniences to you other than the time you spend answering the questions. All data will be anonymous and confidential.

How will my personal information be protected? MTurk protects your anonymity and so it will be impossible for anyone to identify who you are. All the questions we are planning to ask are general, such as age and sex, and will therefore be too general to ever identify anyone.

You must be of legal age to participate in this study.

If you have any questions, please contact the study lead at aaron.reeves@spi.ox.ac.uk

Dr Aaron Reeves, PhD, Oxford University

By clicking "continue," you confirm that you have been informed of the study's goals and procedure in writing, that you have understood this information, and that you are aware that participation is

voluntary. You may abort participation at any point without citing a reason and without incurring any negative consequences as a result of doing so.

Control Condition

[No additional questions]

Treatment #1: Economics

Q6 As you are probably aware, the Coronavirus disease (Covid-19) has spread around the world. Experts are wrestling with the impact of this pandemic on the US economy. Some estimates suggest that the economy could shrink by 3.2% this year, 52.8 million people could end up without work (about 32% of the entire workforce), and that the value of stocks and shares could fall by around 30%.

On the next page, there will be a few factual questions about this information.

Q7 Here are some questions about the information presented on the previous page. Please answer to the best of your ability. If you are not sure, you may return to the previous page. (All answers must be correct in order to proceed in the survey.)

[next page…]

Q8 What is the estimated number of job losses created by Covid-19 in the United States, as stated on the previous page?

- ◯ It was **more than** 50 million (1)
- ◯ It was **less than** 50 million (2)

OR…

Q11 How much is the stock market expected to fall as a result of Covid-19?

- ◯ It was **more than** 20% (1)
- ◯ It was **less than** 20% (2)

Treatment #2: Health

Q12 As you are probably aware, the Coronavirus disease (Covid-19) has spread around the world. Experts are wrestling with the impact of this pandemic on public health in the US. One estimate suggests that around 12.9 million will require hospitalization (3.9% of the population), around 3.7 million will need critical care, and over 2.8 million people will die (around 0.8% of the population). At present, there is no vaccine for Coronavirus and no cure.

On the next page, there will be a few factual questions about this information.

[Next page…]

Q13 Here are some questions about the information presented on the previous page. Please answer to the best of your ability. If you are not sure, you may return to the previous page. (All answers must be correct in order to proceed in the survey).

Q14 What is the estimated number of fatalities from Covid-19 in the United States, as stated on the previous page? (If you are not sure, check back on the previous page).

- ◯ It was **more than** 2 million people (1)
- ◯ It was **less than** 2 million people (2)

OR…

Q15 How many people will require critical care in an Intensive Care Unit?

- ◯ It was **more than** 3 million people (1)
- ◯ It was **less than** 3 million people (2)

Outcomes

Q3 If a national election were held today, on a scale of 0 (sad) to 100 (happy) how would you feel if Trump and the Republican party were reelected?

0 10 20 30 40 50 60 70 80 90 100

Please use sliding scale. ()	

Q4 On a scale of 0 (not important at all) to 100 (extremely important), how important are each of these policy areas for you? (To what extent should the government pay attention to these policy areas?)

0 10 20 30 40 50 60 70 80 90 100

The American economy ()	
Public health in the US ()	

Q5 On a scale of 0 (strongly disagree) to 100 (strongly agree), how much do you agree/disagree with the following statement:

0 10 20 30 40 50 60 70 80 90 100

The government is at fault for allowing the pandemic to spread. ()	

Q24 On a scale of 0 (disagree) to 100 (agree), how do you feel about the following statement:

0 10 20 30 40 50 60 70 80 90 100

My main worry about Coronavirus disease (Covid-19) is the spread of the infection through the US and the possibility that I, or a member of my family, might become infected. ()	

Q37 On a scale of 0 (disagree) to 100 (agree), how do you feel about the following statement:

0 10 20 30 40 50 60 70 80 90 100

Acharya A, *et al. BMJ Global Health* 2020; 5:e004222. doi: 10.1136/bmjgh-2020-004222

My main worry about Coronavirus disease (Covid-19) is its impact on the economy, including the possibility that I or a member of my family might become unemployed or that my investments might lose value. ()	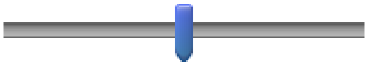

Q23 On a scale of 0 (no responsibility at all) to 100 (total responsibility), how much responsibility does the government bear...

0 10 20 30 40 50 60 70 80 90 100

For the state of the American economy? ()	
For the state of public health in the US? ()	

Background Questions

Q24 In the following questions we ask you to tell us a bit about yourself.

Q25 Gender

- ○ Male (1)
- ○ Female (2)
- ○ Other (please specify) (3) ____

Q26 What year were you born?

▼ 2002 (1) ... 1922 (81)

Q29 How would you describe your current employment?

- ○ Not employed by virtue of retirement, school, or by choice (1)
- ○ Unemployed and looking for work (2)
- ○ Part-time employed (3)
- ○ Full-time employed (4)

Q27 What part of the US do you live in?

▼ Alabama (1) ... Wyoming (81)

Q28 How would you characterize the area where you live?

- Urban (1)
- Suburban/Peri-urban (2)
- Rural (3)

Q30 What is the highest educational level that you have attained, or expect to attain?

- No formal education (1)
- Incomplete primary school (2)
- Complete primary school (3)
- Incomplete secondary school: technical/vocational type (4)
- Complete secondary school: technical/vocational type (5)
- Incomplete secondary school: university-preparatory type (6)
- Complete secondary school: university-preparatory type (7)
- Some university-level education, without degree (8)
- University-level education, with degree (9)

Q31 What is your annual household income? (USD)

- Below $575 per week (or below $30,000 per year) (1)
- Below $960 per week but above $575 per week (or below $50,000 per year but above $30,000 per year) (2)
- Below $1,900 per week but above $960 per week (or below $100,000 per year but above $50,000 per year) (3)

○ Above $1,900 per week (or above $100,000 per year) (4)

Q32 In the previous question, we asked you about your income in general categories. It would be helpful if you could also take a guess at your weekly, monthly, or annual salary (choose one) last year. Please enter a number without commas. (This information is completely confidential).

○ Weekly ($) (1) ____

○ Monthly ($) (2) ____

○ Annual ($) (3) ____

Q33 How much money have you saved for emergencies such as job loss or ill health?

○ Less than a months salary (1)

○ More than a month but less than three months salary (2)

○ More than three months but less than six months salary (3)

○ More than six months salary (4)

Q34 In general, would you say that your health is...

○ Poor (1)

○ Fair (2)

○ Good (3)

○ Very good (4)

○ Excellent (5)

Acharya A, *et al. BMJ Global Health* 2020; 5:e004222. doi: 10.1136/bmjgh-2020-004222

Q35 Have you participated in other surveys focused on Coronavirus?

- Yes (1)
- No (2)

Appendix B: Descriptive Statistics

Table B1: Descriptive statistics for each country sample

	India	UK	US	ALL
Sample Size				
Control *(N)*	501	194	509	1,204
Econ Treatment *(N)*	511	198	529	1,238
Health Treatment *(N)*	487	223	496	1,206
Total *(N)*	1499	615	1534	3648
Total *(%)*	41	17	42	100
Savings equaling consumption of (%)				
Less than a Month	39	41	26	34
Less than 3 Months	35	29	29	32
Less than 6 Months	10	12	20	15
More than 6 Months	16	18	24	20
Self-reported health (%)	39	41	26	34
Poor	4	2	2	3
Fair	11	13	11	11
Good	43	35	36	39
Very Good	29	34	37	33
Excellent	12	16	15	14
Support for incumbent (0-100)				
Control	65	46	47	53
Econ Treatment	66	44	45	52
Health Treatment	65	43	46	52
Total	65	44	46	53
Govt at fault (0-100)				
Control	49	58	61	56
Econ Treatment	50	54	58	54
Health Treatment	51	58	59	55
Total	50	57	59	55

	India	UK	US	ALL
Fear employment disruption (0-100)				
Control	72	59	65	67
Econ Treatment	72	62	62	66
Health Treatment	69	59	61	64
Total	72	60	63	66
Fear contracting Covid (0-100)				
Control	63	70	74	69
Econ Treatment	66	70	73	70
Health Treatment	66	71	73	70
Total	65	71	73	69
Govt can affect economy (0-100)				
Control	76	75	79	77
Econ Treatment	73	76	78	76
Health Treatment	71	73	77	74
Total	74	75	78	76
Govt can affect public health (0-100)				
Control	81	87	83	83
Econ Treatment	81	87	84	83
Health Treatment	81	86	84	83
Total	81	87	84	83
Govt responsible for economy (0-100)				
Control	74	74	74	74
Econ Treatment	71	75	73	73
Health Treatment	72	75	74	73
Total	72	75	74	73
Govt responsible for public health (0-100)				
Control	77	79	77	78
Econ Treatment	77	80	77	78
Health Treatment	78	80	77	78
Total	77	80	77	78

Table B2: Sample characteristics compared to estimates in the underlying population

	India		United Kingdom		United States		Total
	Sample	*Population*	*Sample*	*Population*	*Sample*	*Population*	*Sample*
Sample size							
Total *(N)*	1499	--	615	--	1534	--	3648
Total *(%)*	41	--	17	--	42	--	100
Gender (%)							
Male	73	52	68	49	58	50	66
Female	27	48	31	51	42	50	34
Others	0	-	1	-	0	-	0
Urban/Rural (%)							
Rural	19	66	14	17	14	18	16
Urban, suburban	81	34	86	83	86	82	84
Education (%)							
Completed university	78	11	58	29	61	36	68
Some university	9	3	16	18	11	20	11
No university	13	87	26	47	28	44	21
Income quadrants (%)							
Bottom quartile	12	25	24	25	21	25	18
Second quartile	32	25	25	25	27	25	29
Third quartile	28	25	19	25	35	25	29
Fourth quartile	28	25	31	25	17	25	24
Employment status (%)							
Full-time	65	-	49	62	70	62	65
Retired or no work	5	-	18	23	9	21	9
Part-time	21	-	19	12	13	13	17
Unemployed	9	9	14	3	8	4	9
Age (Median, over 18)	31	35	29	48	37	45	33

Notes: Gender, Urban/Rural distinction in population comes from World Bank Indicators. Education for the UK comes from the European Social Survey from 2018 and for India and the US from the World Values Survey in 2014. Employment status for US comes from Labor Force Statistics from the Current Population Survey, for the UK it comes from the Office for National Statistics and are based on all population above the age 18, for India the unemployment rate comes from Centre for Monitoring Indian Economy as reported. Median age for US, UK, and India comes from latest census projections.

Acharya A, *et al. BMJ Global Health* 2020; 5:e004222. doi: 10.1136/bmjgh-2020-004222

Table B3: Distribution of support for the incumbent across the feeling thermometer by country

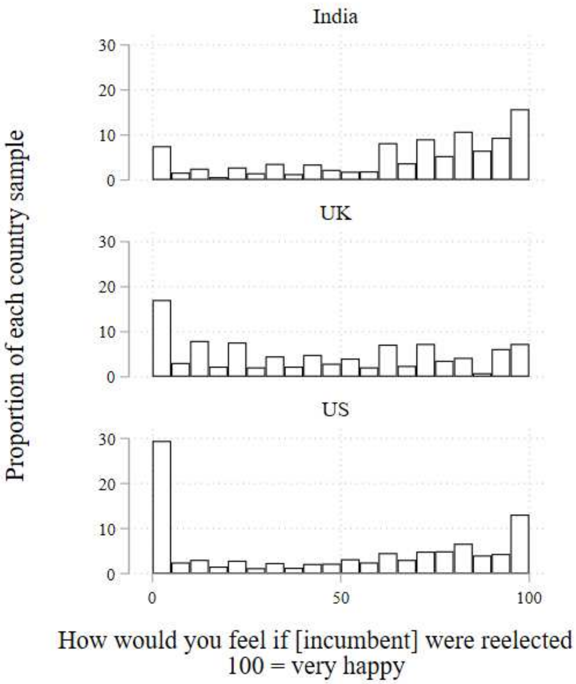

Appendix C: Additional Analyses

Table C1: Government at Fault, Disaggregated by Subgroup

Subgroups	Control	Economic Treatment	Health Treatment	p-value of joint test across treatment conditions
Gender				
Male	55.82	54.11	54.21	0.45
Female	55.04	52.98	57.80	0.07
Urban/Rural				
Rural	51.69	48.49	52.65	0.36
Suburban/Peri-urban	50.96	51.99	52.19	0.83
Urban	59.65	56.43	58.28	0.16
Economic status				
Full-time employed	55.66	54.47	55.64	0.67
Retired	55.12	48.60	54.78	0.21
Part-time employed	54.52	53.33	53.65	0.92
Unemployed	59.23	54.51	59.47	0.37
Education				
University	55.14	52.92	54.51	0.31
Some university	57.98	56.88	57.15	0.95
No university	55.96	54.53	57.29	0.59
Income				
Bottom quartile	59.63	58.49	57.69	0.79
2nd quartile	58.28	57.27	59.51	0.62
3rd quartile	56.94	53.95	55.52	0.40
Top quartile	48.03	46.71	49.08	0.62
Health				
Good/Fair/Poor	57.04	55.39	56.72	0.57
Excellent/very good	54.14	51.62	53.92	0.30
Other Covid-19 survey				
No	51.78	50.60	51.40	0.86
Yes	57.43	55.18	57.24	0.24

Statement: The government is at fault for allowing the pandemic to spread. 0=strongly disagree, 100=strongly agree. Results disaggregated by sub-group. Test statistics are joint-Wald tests of whether each treatment condition is equal to the control condition within each sub-category. We are testing whether the treatments vary by sub-groups. The p-value is for the F-Test.

Table C2: Support for Incumbent, Disaggregated by Subgroup

Subgroups	Control	Economic Treatment	Health Treatment	p-value of joint test across treatment conditions
Gender				
Male	55.28	53.52	52.89	0.35
Female	50.67	52.79	51.87	0.67
Urban/Rural				
Rural	51.75	54.18	54.08	0.73
Suburban/Peri-urban	49.64	48.52	49.03	0.90
Urban	56.63	56.16	53.97	0.35
Economic status				
Full-time employed	54.87	53.17	55.05	0.49
Retired	46.75	53.89	52.23	0.29
Part-time employed	52.69	52.97	48.23	0.28
Unemployed	51.89	51.94	39.24	0.01
Education				
University	52.74	52.40	51.55	0.77
Some university	54.38	50.92	53.34	0.70
No university	56.04	57.00	54.96	0.80
Income				
Bottom quartile	53.54	51.65	50.86	0.71
2nd quartile	54.72	50.87	50.35	0.18
3rd quartile	50.08	53.61	54.17	0.23
Top quartile	56.07	55.54	53.17	0.57
Health				
Good/Fair/Poor	51.99	50.59	50.56	0.70
Excellent/very good	55.37	56.54	54.44	0.59
Other Covid-19 survey				
No	54.35	55.30	52.39	0.49
Yes	53.31	52.26	52.45	0.80

Question: If a national election were held today, would you like to see [incumbent party and party leader] reelected? Results disaggregated by sub-group. Test statistics are joint-Wald tests of whether each treatment condition is equal to the control condition within each sub-category. We are testing whether the treatments vary by sub-groups. The p-value is for the F-Test.

Figure C1: Treatment effects among those with moderate views

One possible explanation for our results is that polarized people will have largely stable responses to our questions irrespective of their exposure to making the economic and the health effects of Covid-19 more salient. It is possible, however, that people with moderate views may be responding to our treatments but these treatment effects are masked by the stable views of the polarized. To test this possibility, we first identify those with moderate views on our political outcomes in the control group—those responding absence of any treatment. We focus on the control group initially because we are interested in identifying those individuals who, in the absence of the treatment, would have had moderate views. We do this by focusing on those who reported a score between 36 and 65 on the feeling thermometer. There are 278 people in this range for the measure of government fault and 230 people for the incumbent measure.

We then use Coarsened Exact Matching to match these people with moderate views in the absence of the treatment with similar individuals who were exposed to either of the treatment conditions. By matching on these individuals, we remove from our analysis those individuals in the treatment conditions who are dis-similar from those with moderate views in the control group. We match on the following variables: on their socio-economic status (low, middle, and high), their age (30 and under and over 30), their health, whether they live in an urban, a peri-urban, or an urban area, their gender, and the country in which they live. There are 406 strata (or unique cells). CEM is usually assessed using a global fit statistic $\zeta 1$ (or L1). This fit statistic tells us how imbalanced the data sets are before the matching procedure (1 = completely separable or no-overlap while 0 = perfectly balanced). In our analysis, before the matching procedure, $\zeta 1$ is 0.62 while after the matching procedure $\zeta 1$ has fallen to basically 0, suggesting there is no imbalance left in the sample.

Our matching procedure identifies 1554 individuals who are matched to 266 individuals with weakly-held views on whether government was at fault (35 people could not be matched from the control group). For the incumbent analysis, our matching procedure identifies 1376 individuals who are matched to 197 individuals with weakly-held views on the incumbent (33 people could not be matched from the control group). We then estimate two OLS regression models (replicating our earlier analysis) for each outcome: one is unadjusted and the other is a ‘doubly robust’ model (i.e., matched sample while controlling for the matching variables).

We find that even among this group of people who seem to have moderate views that our treatments are unable to alter whether they blame government for the spread of the virus or their support for the incumbent.

(a) Incumbent support

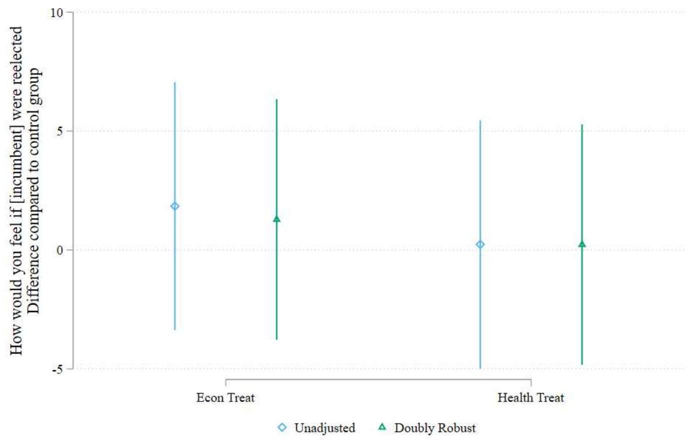

(b) Government at fault

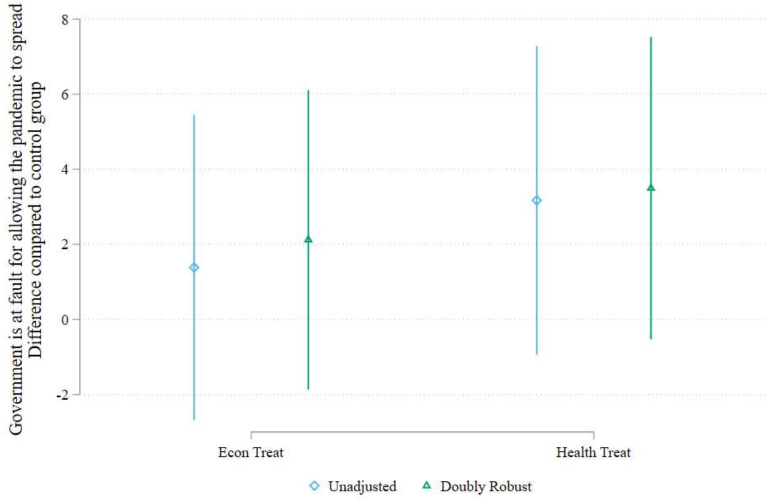

Acharya A, *et al. BMJ Global Health* 2020; 5:e004222. doi: 10.1136/bmjgh-2020-004222

Figure C2: Changes over Time

The US is excluded in these because all surveys were completed within several days, leaving no variation over time to explore.

(a) Incumbent support

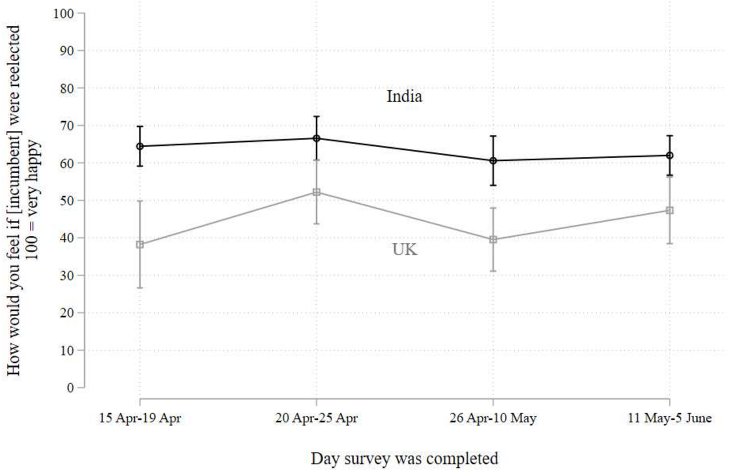

Question: If a national election were held today, would you like to see [incumbent party and party leader] reelected?

(b) Government at fault

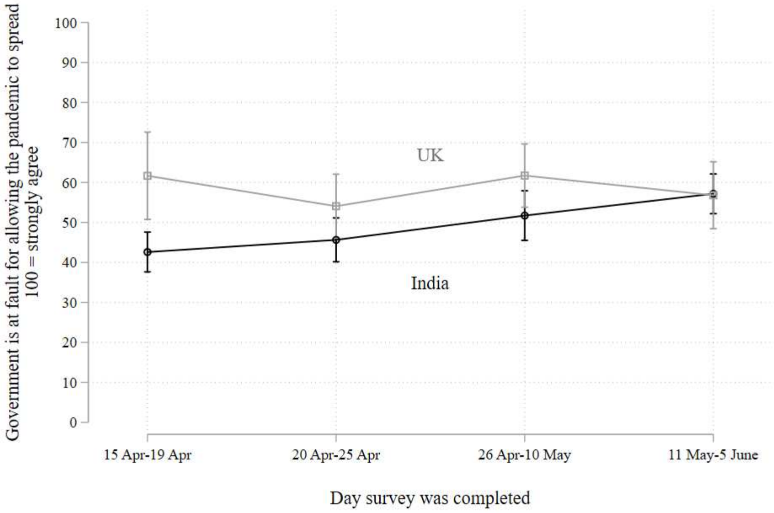

Statement: The government is at fault for allowing the pandemic to spread. 0=strongly disagree, 100=strongly agree.

We also test whether the treatment effect varies according to when people participated in the experiment. We find no clear variation in the treatment effects.

(c) Incumbent support

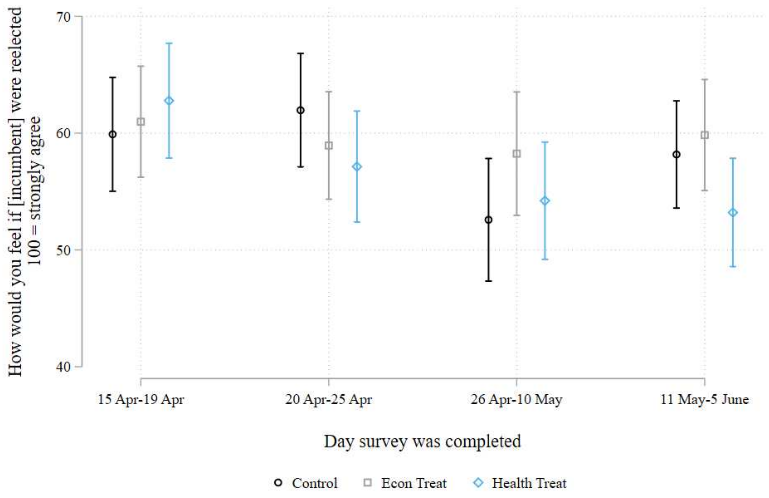

Question: If a national election were held today, would you like to see [incumbent party and party leader] reelected?

(d) Government at fault

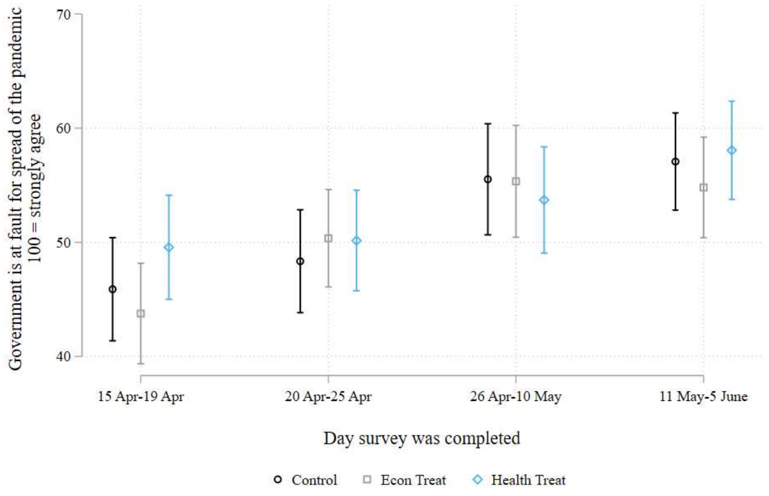

Statement: The government is at fault for allowing the pandemic to spread. 0=strongly disagree, 100=strongly agree.
